# Transient plasma membrane disruption induced calcium waves in mouse and human corneal epithelial cells

**DOI:** 10.1371/journal.pone.0301495

**Published:** 2024-04-17

**Authors:** Zhong Chen, Xiaowen Lu, Mitchell A. Watsky

**Affiliations:** Department of Cellular Biology and Anatomy, Medical College of Georgia, Augusta University, Augusta, GA, United States of America; University of New South Wales, AUSTRALIA

## Abstract

The purpose of this study was to examine transient plasma membrane disruptions (TPMDs) and TPMD-induced Ca^++^ waves (TPMD Ca^++^ Wvs) in human and mouse corneal epithelium (HCEC and MCEC). A multi-photon microscope was used to create laser–induced TPMDs in single cultured cells and in intact *ex vivo* and *in vivo* MCECs and *ex vivo* human cornea rim HCECs. Eye rubbing-induced TPMDs were studied by gentle rubbing with a cotton tipped applicator over a closed eyelid in *ex vivo* and *in vivo* MCECs. Ca^++^ sources for TPMD-induced Ca^++^ waves were explored using Ca^++^ channel inhibitors and Ca^++^-free media. TPMDs and TPMD Ca^++^ Wvs were observed in all cornea epithelial models examined, often times showing oscillating Ca^++^ levels. The sarcoplasmic reticulum Ca^++^ ATPase inhibitors thapsigargin and CPA reduced TPMD Ca^++^ Wvs. TRP V1 antagonists reduced TPMD Ca^++^ Wvs in MCECs but not HCECs. Ca^++^-free medium, 18α-GA (gap junction inhibitor), apyrase (hydrolyzes ATP), and AMTB (TRPM8 inhibitor) did not affect TPMD Ca^++^ Wvs. These results provide a direct demonstration of corneal epithelial cell TPMDs and TPMDs in *in vivo* cells from a live animal. TPMDs were observed following gentle eye rubbing, a routine corneal epithelial cell mechanical stress, indicating TPMDs and TPMD Ca^++^ Wvs are common features in corneal epithelial cells that likely play a role in corneal homeostasis and possibly pathophysiological conditions. Intracellular Ca^++^ stores are the primary Ca^++^ source for corneal epithelial cell TPMD Ca^++^ Wvs, with TRPV1 Ca^++^ channels providing Ca^++^ in MCECs but not HCECs. Corneal epithelial cell TPMD Ca^++^ Wv propagation is not influenced by gap junctions or ATP.

## Introduction

It has long been recognized that small, repairable, plasma membrane tears, termed transient plasma membrane disruptions (TPMDs), frequently occur in a host of different cell types, including GI cells, myocytes, and osteocytes [1–5]. TPMDs are a common form of cell injury created by transient micro-tears in the cell membrane that can result from a variety of insults, ranging from mechanical stresses and strains to bacterial toxin-induced membrane lesions. They must be quickly repaired for the cell to avoid lysis and survive.

While many studies have examined the mechanisms of TPMD repair [6, 7], it has only recently been shown that TPMDs can act as a mechanosensory signaling pathway. TPMDs result in extracellular Ca^++^ influx into the cell at the site of membrane disruption, and trigger a number of signaling events both intra- and extra-cellularly, including intercellular Ca^++^ wave activity (TPMD-Ca^++^ Wvs). TPMD-Ca^++^ Wv related mechanotransduction differs from mechanosensitive ion channel initiated Ca^++^ waves, which have been widely studied (including in cornea cells (e.g. [8]), in that a healable plasma membrane micro-tear initiates the initial intracellular calcium pulse as opposed to ion channels. It was found that osteocyte TPMDs occur in bone under normal mechanical loading conditions, leading to the hypothesis that the TPMD-Ca^++^ Wvs act as a mechanosensory response that coordinates activities of other bone cells, one of the primary functions of osteocytes [3, 9, 10]. Given that TPMDs and their associated Ca^++^ waves are initiated in bone-encased osteocytes via bone mechanical loading, we hypothesize that a similar or more extreme response will occur in cornea epithelial cells (CECs) during routine corneal mechanical loading within the much more flexible and exposed cornea. Such stressors could include external dry eye, particulate matter and mechanical loading associated with blinking, eye rubbing, poking, contact lenses, etc. Our group recently published a study demonstrating the formation of TPMDs in keratocytes following eye rubbing in mouse and human ex *vivo* corneas [11]. That study also determined that intra-and extracellular Ca^++^ act as sources for human and mouse stromal cell TPMD-Ca^++^ Wvs, and that ATP plays a role in the propagation of TPMD-Ca^++^ Wvs in human but not mouse stromal cells, while gap junctions play a small role in mouse but not human TPMD-stromal cell Ca^++^ Wv propagation [11]. The current study examines these phenomena in mouse and human CECs.

## Materials and methods

### Materials

Reagents and the concentrations used were selected from previous TPMD literature [3, 11–14] and from the TRP channel literature [15]. Dulbecco’s modified Eagle’s medium (DMEM) and Ca^++^-free Keratinocyte-SFM medium (K-SFM), and alexa fluor 488 dextran (FDx) were purchased from ThermoFisher Scientific, Waltham, MA. Cal-520-AM dye was purchased from AAT Bioquest, Sunnyvale, CA. Cyclopiazonic acid (CPA), 18-α glycyrrhetinic acid (18α-GA), apyrase, AMG 9810, x-methyltryptophan (AMTB) and ryanodine were purchased from Sigma-Aldrich, St. Louis, MO. Thapsigargin was purchased from Abcam, Cambridge, United Kingdom, and N-(4-tertiarybutylphenyl)-4-(3-cholorphyridin-2-yl) tetrahydropyrazine-1(2H)-carbox-amide (BCTC) was purchased from Focus Biomolecules, Plymouth Meeting, PA.

### TPMDs and TPMD-Ca^++^ Wvs in cultured primary CECs

Primary mouse and human CECs were isolated and cultured from 4 week-old C57BL/6 mouse corneas, and de-identified donor human corneal rim tissue, respectively, as previously described [16–18]. For all studies requiring mouse cells and tissue, mice were sacrificed by CO_2_ overdose followed by cervical dislocation. Isolated CEC were seeded onto 35 mm plates 24h prior to experiments and loaded with the calcium indicator Cal-520-AM (0.25 μM) [11, 19]. Various treatments with appropriate controls (Table 1) were introduced 30–60 min. before the TPMD procedure to determine the source of Ca^++^ for the TPMD-Ca^++^ Wvs along with their mechanism of transmission as described previously for keratocytes [11, 19].

**Table 1:** Summary of experimental treatment results from the current corneal epithelial cell study compared to those from a previous study examining corneal stromal cells [11].

	Mouse CEC		Mouse CSC**		Human CEC		Human CSC**	
Treatment	Number	Area	Number	Area	Number	Area	Number	Area
**K-SFM**	NS	↑	↓	↓	NS	NS	↓	↓
Ca^++^ free medium								
**Cyclopiazonic acid (10 uM, 1h)**	↓	↓	N/A	N/A	↓	↓	N/A	N/A
Inhibits SERCA								
**Thapsigargin (1 uM, 1h)**	↓	↓	↓	NS	↓	↓	↓	NS
Depletes intracellular Ca^++^								
**Thapsigargin (1 uM, 1h) + K-SFM** ^**#**^	↓	↓	↓	↓	↓	↓	↓	↓
**18α-GA (30 uM, 1h)**	NS	NS	NS	↓	NS	NS	NS	NS
Inhibits gap junctions								
**Apyrase (10 U/ml, 30 min.)**	↑	NS	NS	NS	NS	NS	↓	↓
Hydrolyzes ATP								
**BCTC (10 uM, 1h)**	↓	NS	NS	NS	NS	NS	NS	NS
Inhibits TRPV1 and TRPM8								
**AMG 9810 (10 uM, 30 min.)**	↓	↓	↓	NS	NS	NS	NS	NS
Inhibits TRPV1								
**AMTB (10 uM, 30 min.)**	NS	NS	NS	NS	NS	↑	NS	NS
Inhibits TRPM8								
**Ryanodine (50 uM, 1h)**	N/A	N/A	N/A	N/A	NS	↓	N/A	N/A
Depletes intracellular Ca^++^								
**Ryanodine (50 uM, 1h) + K-SFM** ^**#**^	↓	↓	↑	↓	NS	↓	↓	↓

* Compared to DMEM control

# Compared to K-SFM

** Data cited from reference 11.

NS: not significantly different compared to DMEM

N/A: not available

TPMDs were produced and analyzed as described for keratocytes [11]. Briefly, a single cell was targeted using the single-time bleaching setting of a Zeiss 780 upright multi-photon microscope. Cal-520-AM fluorescence was recorded under a 20x objective using a 488 nm laser. 5–10 source cells per culture plate were individually targeted for TPMDs, and each experiment included at least 3 plates. Criteria for targeting cells included morphological appearance (healthy cells) and nearby neighbor cells, with all cells dye loaded. Cells were considered positive responders only when they increased their baseline fluorescent intensity by a minimum of 50%. Both the number of positively responding cells and area under the fluorescence versus time curve were measured. The number of positively responding cells represents the general spread of the response. The under curve area value equates to the magnitude and time course of the response and is the more sensitive measure in that it determines the response level over time rather than only the number of cells responding. This value was determined by measuring and averaging the area under the fluorescent intensity vs. time curve for individual cells that reached the defined intensity change threshold. DMEM containing Ca^++^ was used as control.

### TPMDs and TPMD-Ca^++^ Wvs in mouse corneas and human corneal rim tissue

C57BL/6 mice were sacrificed and globes were removed and placed for 3h in DMEM solution containing Cal-520 AM (75 uM). After washing with PBS, whole globes were placed onto a silicone pad, epithelial side up, and TPMDs were created in individual cells within the multilayered CE with the multiphoton microscope. For human corneal rims, Cal-520-AM (5 μM) loading was performed as described above. After washing with PBS, the Cal-520-AM loaded corneal rims were pinned onto a silicone pad epithelial side up and the 820 nm laser was used to produce a TPMD in a single targeted cell.

For live mouse studies, animals were anesthetized with ketamine/xylazine and topical proparacaine, and Cal-520 AM dropped onto the eye over a 1h period. Anesthetized mice were situated under the Zeiss multiphoton microscope and the CE brought into focus. TPMDs were created in single cells as in the *ex vivo* CE studies. All mouse studies conformed to the ARRIVE guidelines, the ARVO statement for the use of animals in ophthalmic and vision research, and were approved by the Augusta University IACUC.

### TPMDs after cornea rubbing

To generate CE TPMDs, mice were anesthetized with ketamine/xylazine and topical proparacaine, PBS containing 2 mg/ml FDx was dropped on the cornea, and a moistened cotton tipped applicator was gently rubbed over the closed lid five times. FDx can only enter and remain in cells that have had plasma membrane disruptions that subsequently healed, and is visible by its fluorescent signal. Thus FDx is a marker for cells that have had TPMDs [3, 11]. After FDx application, the eye was rinsed 3 times with PBS, and 5 μl 4mg/ml propidium iodide was dropped onto the cornea as a dead cell indicator and the cornea was allowed to sit for 30 minutes before rinsing 3 times with PBS. Mice were sacrificed and globes were removed and placed in 4% paraformaldehyde (PFA) on ice for 75 minutes.

Corneas were isolated from the fixed globe and 4 radial incisions were made to allow for flat-mounting on a microscope slide. Mounting solution (Fluoroshield™ with DAPI, Sigma-Aldrich, St Louis) was dropped onto the cornea which was placed under a coverslip for CEC imaging with a Leica Stellaris Confocal Microscope (Danaher Corporation, Washington, D.C.). FDx positive and DAPI stained cells were quantified using the Imaris image processing software Spots package (Oxford Instruments, Abingdon, UK). By selecting the average diameter of the cells and setting a fixed initial sensitivity, the software labels individual cells as spots and reports a cumulative spots count for each image. After an initial rough pass in which the count was completely automated by the software, overexposed and underexposed areas were accounted for by manual spot deletion or addition. A conservative approach was taken such that only clear cells (spots) in heavily saturated parts of the image were counted.

### Statistical analysis

Data are provided as the mean ± SE of at least three experiments. Differences between DMEM controls and treatment groups were compared using the Student’s t-test.

## Results

### Single cell-induced TPMD-Ca^++^ Wvs in CECs

**Fig 1:**
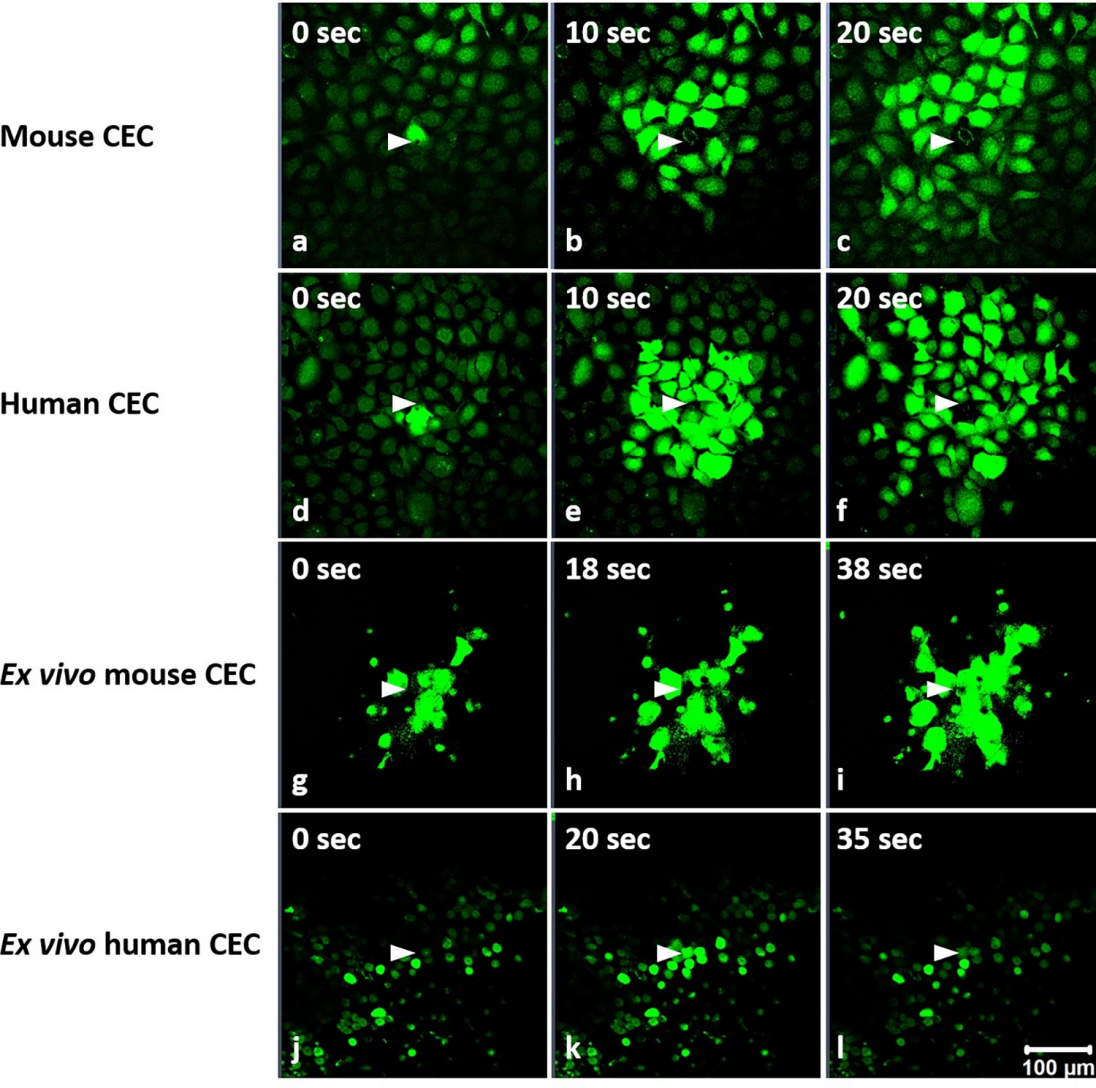
Multi-photon laser induced TPMD-Ca^++^ Wvs in cultured and *ex vivo* mouse and human CECs. Representative TPMD-Ca^++^ Wv images of cultured mouse (**a**-**c**) and human CECs (**d**-**f**), along with *ex vivo* mouse corneal rim (**g**-**i**) and human CECs (**j**-**l**). TPMD-Ca^++^ Wvs are shown at different time points following a single cell TPMD. White arrows point to the TPMD initiation site; other cells were not wounded.

**Fig 2:**
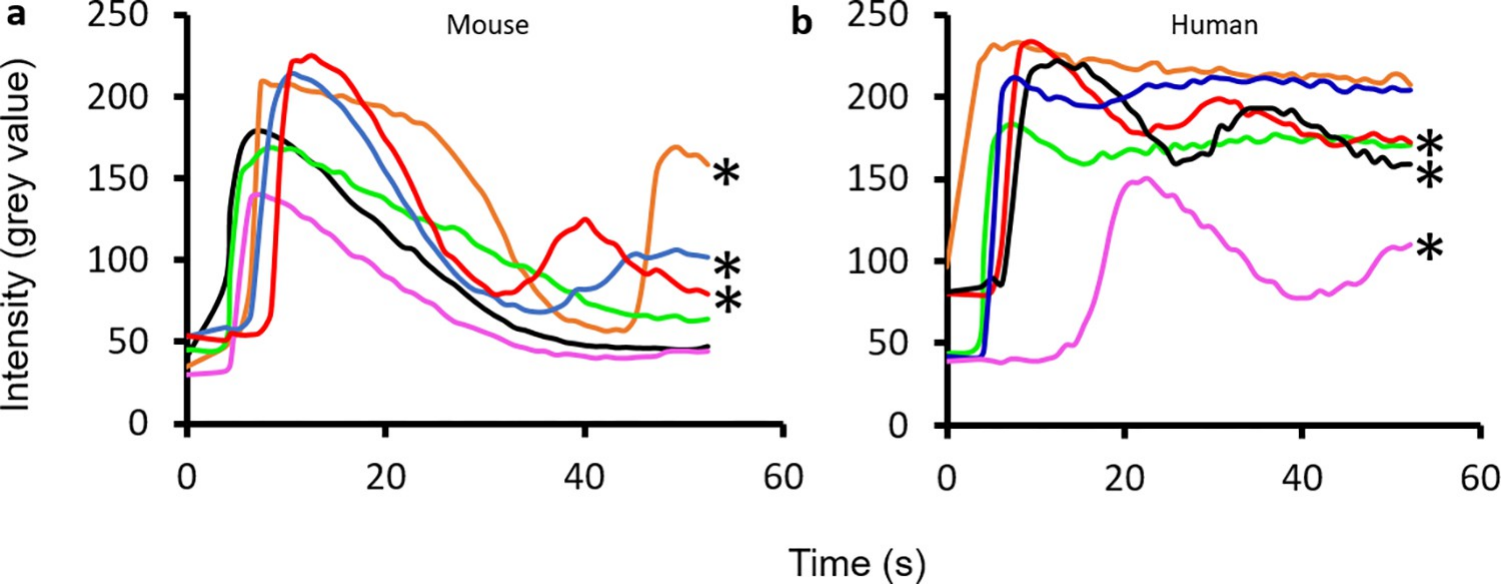
Representative fluorescence intensity curves of select individual (a) mouse and (b) human CEC within a single culture dish visual field. Colors represent different cells. * marks curves showing Ca^++^ level oscillations within the cell. Most cells in the dishes did not show this cycling. TPMD was created at time 0.

Single cell TPMDs created with the multi-photon laser resulted in TPMD-Ca^++^ Wvs in cultured primary mouse and human CECs, intact *ex vivo* mouse CECs, and fresh human corneal rim CECs (Fig 1, S1–S4 Figs (videos)). Where mouse cornea epithelial cells are typically smaller than human cells, the *ex vivo* human CEC shown in Fig 1J–1L are smaller than their mouse counterparts because the mouse cells are from the superficial epithelium, which are the largest cornea epithelial cells, and the human CEC are from basal layers due to cell loss that occurs during storage of the cornea rims for transplant. The storage medium is designed to preserve corneal endothelial cells, and it is common to lose the upper epithelial layers during storage. In addition to TPMD-Ca^++^ Wvs, several cells in each image field (although not the majority of cells) also demonstrated oscillating Ca^++^ levels at a frequency of approximately 0.03–0.05Hz, as illustrated in Fig 2.

### CEC TPMD-Ca^++^ Wvs in live mice

**Fig 3:**
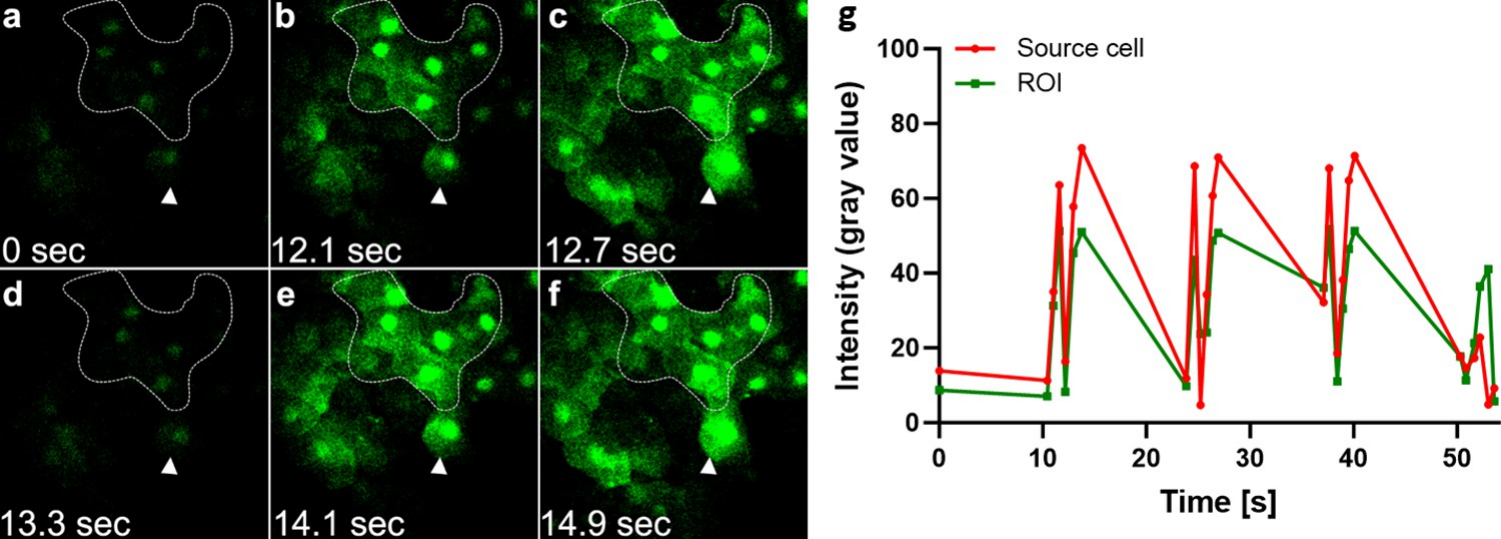
Representative multiphoton laser-induced TPMD-Ca^++^ Wv and subsequent Ca^++^ level cycling in a living mouse cornea. **a**-**f**. TPMD-Ca^++^ Wv spread at 0–14.9s following TPMD initiation in a single source cell (arrowhead). Note the oscillating Ca^++^ level in the outlined region of interest (ROI). **g**. Graph of the fluorescence intensity of the source cell and ROI for 54s following TPMD-Ca^++^ Wv initiation. TPMD created at time 0.

To demonstrate that TPMD-Ca^++^ Wvs occur in live mouse CECs, multiphoton laser-induced single cell TPMDs were produced in Cal-520 AM pre-loaded CEC of anesthetized mice, and TPMD-Ca^++^ Wvs were visualized as in the *ex vivo* CECs. Fig 3 and S5 Fig (video) show a representative laser-induced TPMD-Ca^++^ Wv in a living mouse cornea. The arrowhead in Fig 3 points to the source cell that the TPMD was created in. In addition to the TPMD-Ca^++^ Wv, Ca^++^ level oscillations were observed in the source cell and in many of the cells that experienced the Ca^++^ Wv. Similar results were routinely observed in other living mouse corneas examined (n = 5).

### Creation of CEC TPMDs in intact mouse corneas by rubbing

**Fig 4:**
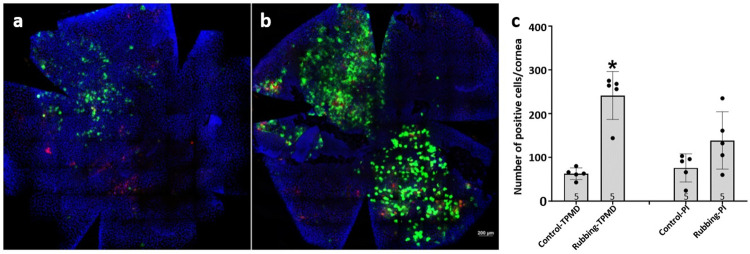
CEC TPMDs in intact mouse corneas before (a) and after (b) rubbing 5 times over a closed lid. (c) IMARIS cell counting of TPMD and PI positive cells per the total number of cells in control and rubbed corneas (n = 5 each). Blue indicates DAPI stained nuclei (total cell number), green indicates TPMD (FDx) positive cells, and red indicates PI positive (dead) cells. Black dots in c show cell number ratios in the individual corneas examined. * indicates P<0.05 when compared to control.

CEC TPMDs were observed in intact mouse corneas following rubbing over the closed lid of anesthetized mice. FDx was applied to the cells to observe TPMD formation, and PI was added to observe any cell death that might have been created by the rubbing event. Prior to rubbing a small number of TPMD positive epithelial cells can be seen (Fig 4A and 4C), presumably resulting from normal corneal mechanical stressors. After rubbing 5 times, significantly more positive TPMD cells were observed (Fig 4B and 4C). A small number of PI positive cells were observed in the superficial epithelium, with no significant difference between control unrubbed and rubbed corneas (Fig 4A–4C).

### Mouse CEC TPMD-Ca^++^ Wv initiation and propagation

**Fig 5:**
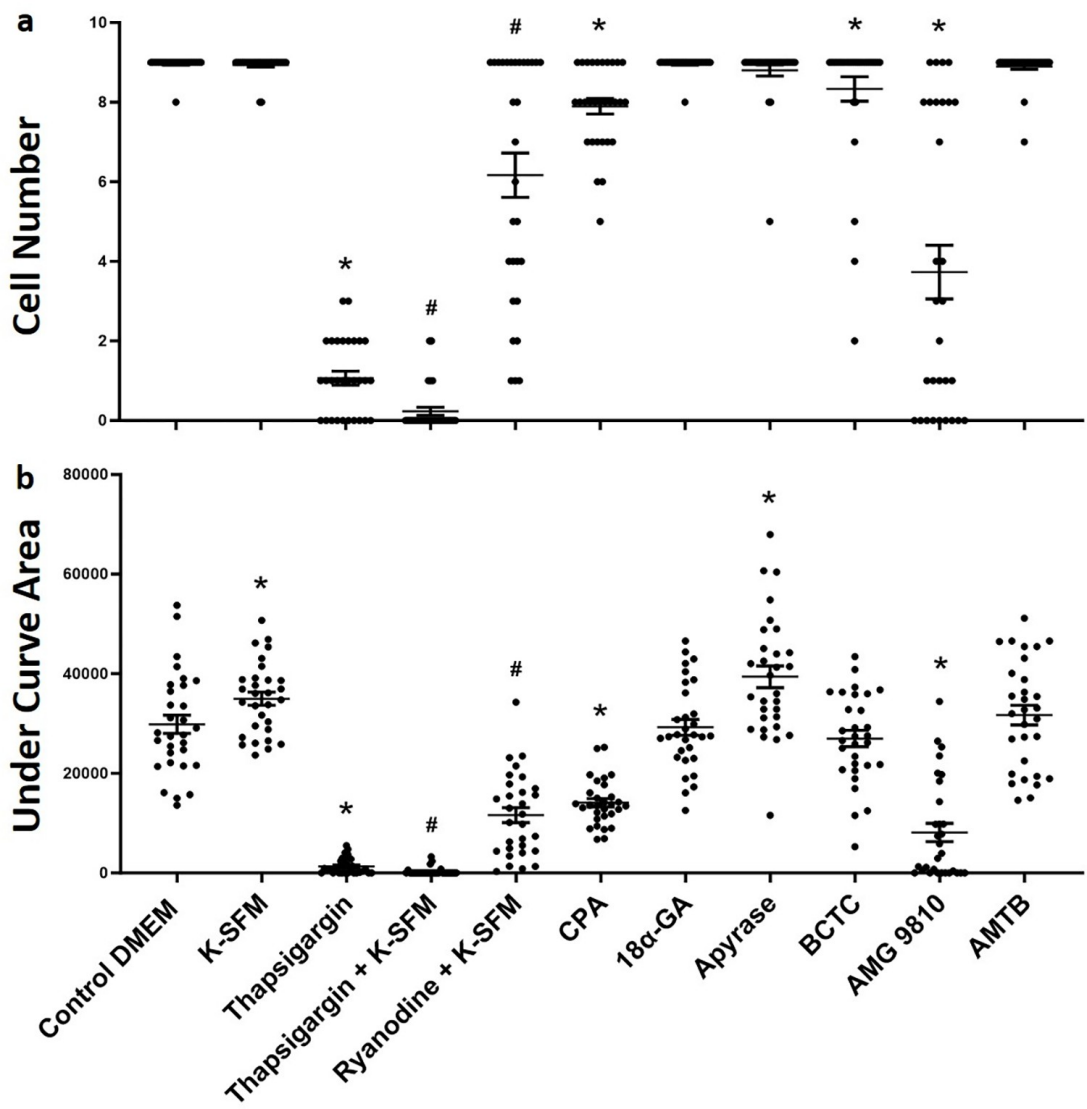
Cell number (a) and under curve area (b) of TPMD induced Ca^++^ signaling in mouse CECs. All compounds added to DMEM execpt for those indicated in K-SFM, which was Ca^++^ free. Individual data points indicate number of cells measured from 3 individual culture plates (10 cells per plate). Data presented as mean ± SE. * indicates P<0.05 when compared to control DMEM; # indicates P<0.05 when compared to K-SFM.

In order to determine the Ca^++^ source and propagation signaling mechanism of TPMD-Ca^++^ Wvs in mouse CECs, various culture medium formulations and pharmaceutical agents were utilized (Table 1). Addition of Ca^++^ free K-SFM medium to the cells prior to initiating TPMDs was used to determine the dependence of extracellular Ca^++^ on TPMD-Ca^++^ Wvs. Ca^++^ free K-SFM had no effect the TPMD-Ca^++^ Wv cell number value, and, interestingly, it increased the area under the curve (under curve area) (34986 ± 1322) when compared to control DMEM (29849 ± 1849) (P<0.05) (Fig 5).

Thapsigargin and ryanodine application to cells in both Ca^++^-free K-SFM and Ca^++^-containing DMEM was utilized to determine the role of intracellular Ca^++^ on TPMD-Ca^++^ Wvs. The cell numbers of thapsigargin in K-SFM (0.23 ± 0.10), ryanodine in K-SFM (6.16 ± 0.55), and under curve areas of thapsigargin in K-SFM (292 ± 143) and ryanodine in K-SFM (11605 ± 1504), were significantly reduced when compared to the K-SFM alone cell number (8.93 ± 0.25) and under curve area (34986 ± 7246) (both P<0.05), respectively (Fig 5).

Adding thapsigargin to DMEM, where the DMEM will allow for extracellular Ca^++^ to affect TPMD-Ca^++^ Wvs, the TPMD-Ca^++^ Wvs were also significantly reduced when examining both the cell number (1.06 ± 0.17) and under curve area (1314 ± 291) as compared to control DMEM (8.96 ± 0.03 and 29849 ± 1849, respectively; P<0.05) (Fig 5). CPA, another sarcoplasmic reticulum Ca^++^ ATPase inhibitor, was used as an additional compound to examine the role of intracellular Ca^++^ on TPMD-Ca^++^ Wvs. CPA significantly reduced TPMD-Ca^++^ Wv cell number (7.90 ± 0.19) and under curve area (14092 ± 838) when compared to control DMEM (P<0.05), respectively (Fig 5). Fig 7 shows representative mouse CEC TPMD-Ca^++^ Wvs following thapsigargin treatment.

18α-GA, a gap junction inhibitor, and apyrase, a compound that hydrolyzes ATP, were used to analyze the influence of gap junctions and ATP release on TPMD-Ca^++^ Wv propagation, respectively. Neither compound reduced the cell number or under curve area, with apyrase application actually increasing the under curve area (39389 ± 2195) when compared to control DMEM (P<0.05) (Fig 5).

AMG9810, a TRPV1 inhibitor, AMTB, a TRPM8 inhibitor, and BCTC, an inhibitor of both the transient receptor potential vanilloid 1 (TRPV1) and transient receptor potential melastatin 8 (TRPM8) ion channels were used to examine the role of TRPV1 and TRPM8 on TPMD-Ca^++^ Wv propagation. AMG9810 significantly reduced the TPMD induced Ca^++^ signaling for both cell number (3.73 ± 0.67) and under curve area (8128 ± 1826) when compared to control DMEM (P<0.05). AMTB had no effect on TPMD-Ca^++^ Wv propagation, while BCTC reduced cell number (8.33 ± 0.30) when compared control DMEM (P<0.05) (Fig 5). Fig 7 shows representative mouse TPMD-Ca^++^ Wvs following AMG9810 treatment. A summary of all experimental treatment results in mouse CECs is presented in Table 1.

### Human CEC TPMD-Ca^++^ Wv initiation and propagation

**Fig 6:**
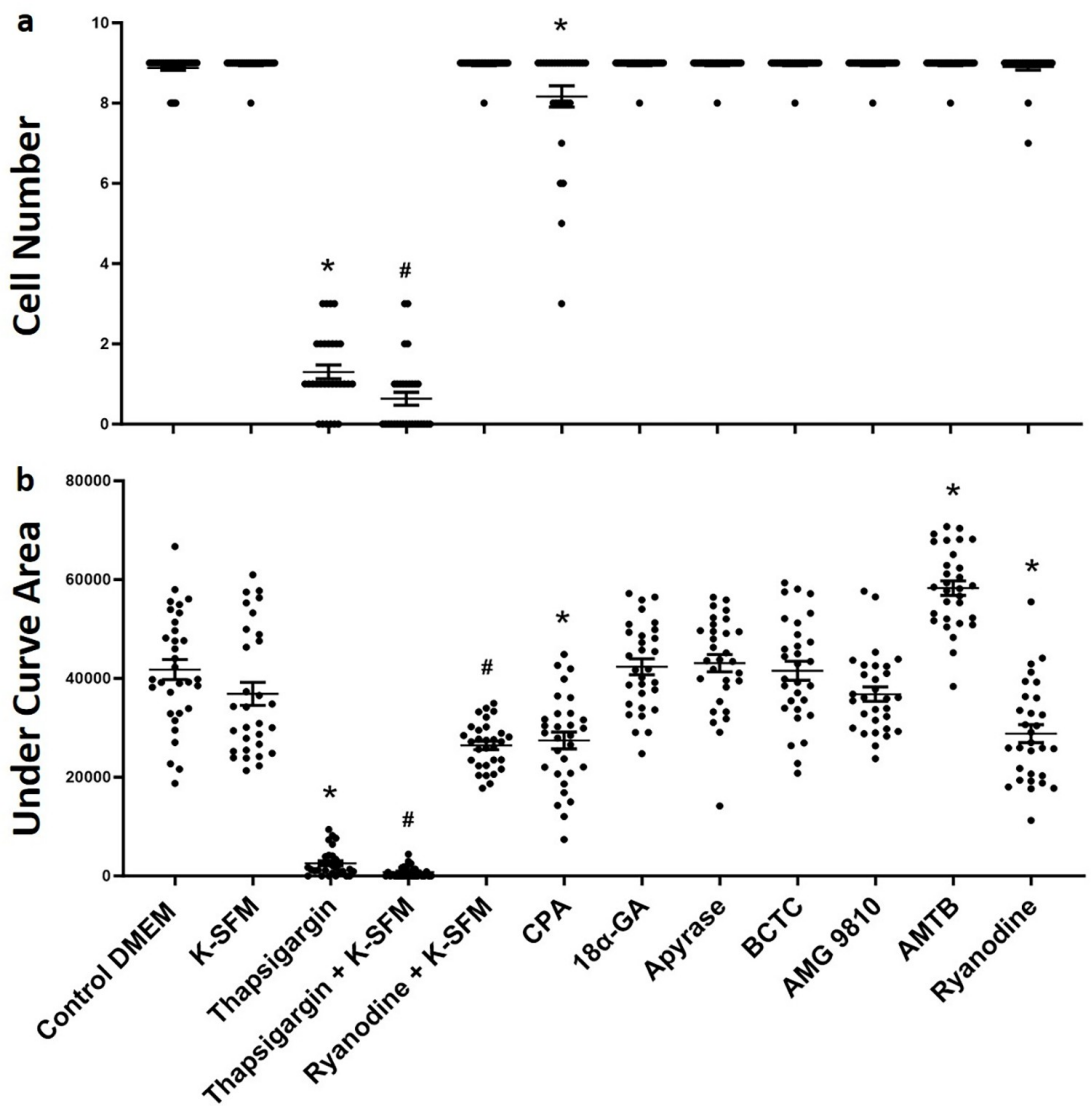
Cell number (a) and under curve area (b) of TPMD induced Ca^++^ signaling in human CECs. All compounds added to DMEM execpt for those indicated in K-SFM, which was Ca^++^ free. Individual data points indicate number of cells measured from 3 individual culture plates (10 cells per plate) except for the control DMEM group, which used 6 culture plates (32 cells examined). Data presented as mean ± SE. * indicates P<0.05 when compared to control DMEM; # indicates P<0.05 when compared to K-SFM.

As in the mouse CECs, various culture medium formulations and pharmaceutical agents were utilized to determine the Ca^++^ source(s) and propagation signaling mechanism of human CEC TPMD-Ca^++^ Wvs (Table 1). Similar to the mouse results, Ca^++^ free K-SFM had no effect on human CEC TPMD-Ca^++^ Wvs (Fig 6).

**Fig 7:**
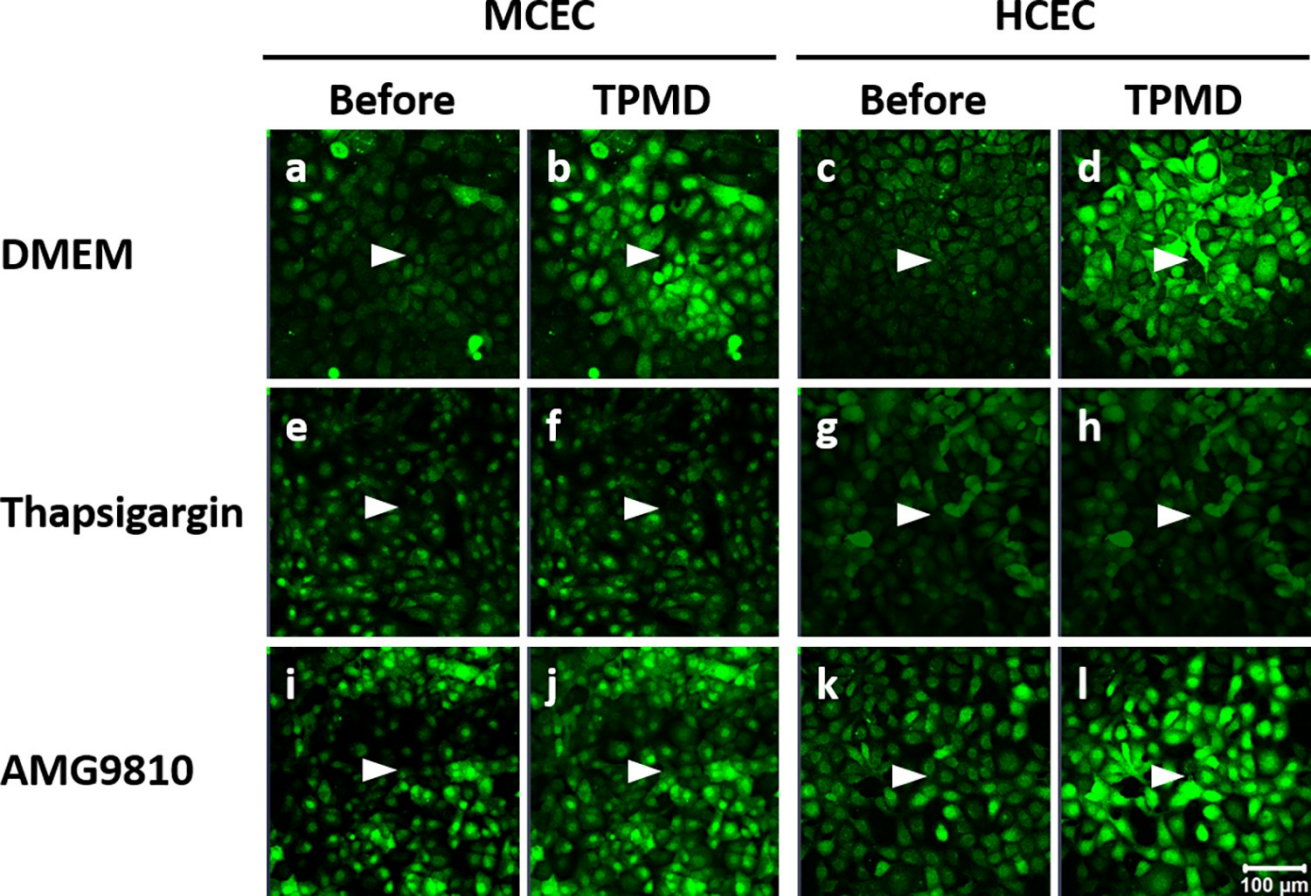
Influence of thapsigargin and AMG9810 treatment on TPMD-Ca^++^ Wvs compared to control DMEM in mouse and human CECs. Representative images of primary mouse and human CECs are presented showing cells before and after laser induced TPMD initiation. White arrows show the TPMD source cell in each image. No other cells were wounded.

Similar to mouse CECs, intracellular Ca^++^ stores were important for generating human CEC TPMD-Ca^++^ Wvs. Thapsigargin applied to human CECs in Ca^++^ free K-SFM resulted in lower TPMD-Ca^++^ Wv activity, reducing both the cell number (0.63 ± 0.16) and under curve area (764 ± 209), compared to control DMEM cell number (8.87 ± 0.05) and under curve area (41792 ± 2030) (P<0.05). Treating with ryanodine + K-SFM resulted in reduction of the under curve area (26427 ± 2505), but not cell number (P<0.05), when compared to K-SFM cell number (8.96 ± 0.03) and under curve area (36878 ± 2340). Thapsigargin in DMEM significantly reduced both cell number (1.30 ± 0.17) and under curve area (2573 ± 494) (both P<0.05), when compared to respective control DMEM values (see Fig 6). Ryanodine in DMEM medium significantly reduced only the under curve area (27503 ± 143) (P<0.05), when compared to control DMEM (see Fig 6). CPA significantly reduced the cell number (8.16 ± 0.26) and under curve area (27439 ± 1699) (both P<0.05), when compared to control DMEM (Fig 6). Fig 7 and S6–S9 Figs (videos) show representative human CEC TPMD-Ca^++^ Wvs following thapsigargin treatment.

Examining the influence of gap junctions and extracellular ATP on TPMD-Ca^++^ Wv propagation, neither 18α-GA or apyrase, respectively, had any influence on the cell number or under curve area values (Fig 6). Unlike mouse CECs, AMG9810, AMTB, and BCTC did not reduce TPMD-Ca^++^ Wv propagation in human CECs, indicating no influence of TRPV1 or TRPM8 on this activity. AMTB actually resulted in an increased under curve area (58270 ± 1477) compared to the control DMEM (P<0.05) (Fig 6). Fig 7 and S6–S9 Figs (videos) show representative TPMD-Ca^++^ Wvs in mouse and human CEC following thapsigargin and AMG9810 treatment. A summary of all experimental treatment results in human CECs can be seen in Table 1.

## Discussion

CECs are located on the anterior surface of the cornea, function as the primary physical barrier to outside environmental risk factors, and serve important functions related to visual acuity, corneal health and wound healing. They are active cells with a rapid turnover process. Direct exposure of CECs to the external environment make them prone to environmental-related mechanical stressors, and therefore extremely vulnerable to TPMDs. Previous studies have linked corneal epithelial Ca^++^ Wvs to the cornea wound healing response [20, 21].

Our group recently demonstrated that TPMDs can be produced in keratocytes by corneal rubbing, a common mechanical stressor, in both human corneal rims and mouse corneas [11]. We also demonstrated TPMD-Ca^++^ Wvs initiated by laser-induced single cell TPMDs in corneal keratocytes as well as in *ex vivo* human corneal rims and mouse corneas. In addition to keratocytes, TPMDs, and in some cases TPMD-Ca^++^ Wvs, have previously been shown to occur in several cell types including gastrointestinal tract cells, myocytes and osteocytes [1–4]. The current study demonstrates that like keratocytes, TPMDs can be produced in mouse CECs by cornea rubbing, which resulted in no concurrent increase in cell death. The current study also demonstrates TPMD-Ca^++^ Wvs initiated following creation of a TPMD in a single cell of mouse and human CECs, and in a live mouse cornea. In addition, Ca^++^ level oscillations were observed in a subset of epithelial cells that had experienced TPMD-Ca^++^ Wvs. Similar results have previously been reported for corneal epithelial cells experiencing Ca^++^ Wvs following scratch wounding or scraping, although at a much lower frequency than observed following TPMD initiation [20]. These previous epithelial wound models were much more traumatic than the TPMDs examined in the current study.

While extracellular Ca^++^ was shown to play an important role in TPMD-Ca^++^ Wv proliferation in keratocytes [11], the failure of Ca^++^ free medium to influence TPMD-Ca^++^ Wvs in the current study indicates that mouse and human CECs do not require extracellular Ca^++^ for initiation or proliferation of TPMD-Ca^++^ Wvs. On the other hand, as with keratocytes [11], the reduction of TPMD-Ca^++^ Wv activity in CECs following disruption of intracellular Ca^++^ transport and release indicates a significant reliance on intracellular Ca^++^ for TPMD-Ca^++^ Wv initiation and propagation. Thapsigargin and CPA, which inhibit sarcoplasmic reticular Ca^++^-ATPase, significantly reduced TPMD-Ca^++^ Wv cell number and under curve area in both mouse and human CECs. Similarly, ryanodine, which binds to ryanodine receptors resulting in depletion of the intracellular Ca^++^ stores, also reduced TPMD-Ca^++^ Wv cell number and under curve area in mouse and human CECs. Thapsigargin and ryanodine were also added to Ca^++^ free K-SFM to eliminate the influence of extracellular Ca^++^, and both significantly reduced mouse and human TPMD-Ca^++^ Wv cell number and under curve area, although ryanodine + K-SFM failed to reduce the TPMD-Ca^++^ Wv cell number in human CECs as compared to K-SFM alone. This could indicate a small reliance of extracellular Ca^++^ on human CEC TPMD-Ca^++^ Wvs. In addition, we previously noted that under area curve is the more sensitive marker for TPMD-Ca^++^ Wv activity [11], and this current result is in agreement with those previous findings.

ATP is a major cellular energy source and plays an important role in initiating and regulating Ca^++^ signaling. ATP influences CSC TPMD-Ca^++^ Wv initiation [11], and has also been shown to initiate TPMD-Ca^++^ Wvs in osteocytes [3]. Apyrase, which hydrolyzes ATP, had no effect on mouse or human CEC TPMD-Ca^++^ Wvs indicating no ATP effect on CEC TPMD-Ca^++^ Wvs. This is in contrast to human (but not mouse) keratocytes [11], where apyrase inhibited both TPMD-Ca^++^ Wv cell number and area under curve.

Cornea keratocytes communicate with one another through gap junctions [22], and gap junctions were shown to have a small influence on mouse (but not human) CSC TPMD-Ca^++^ Wvs [11]. 18α-GA, a gap junction inhibitor, had no influence on mouse or human CEC TPMD-Ca^++^ Wvs, indicating that gap junctions are not involved in CEC TPMD-Ca^++^ Wv initiation or propagation.

Transient receptor potential (TRP) channels are non-selective cation channels with variable Ca^++^ ion selectivity. While TRPV1 and TRPM8 are the only calcium transporters described to date in keratocytes, we recently demonstrated that neither play a role in regulating TPMD-Ca^++^ Wvs in human CSC, while TRPV1 appears to play a small role in mouse CSC signaling [11]. Mammalian CEC Ca^++^ channels identified to date include TRPV1, V3, V4, and M8, and a PLA2-sensitive Ca^++^ channel [23–29]. The current study utilized the TRP channel blockers BCTC (an inhibitor of both TRPV1 and TRPM8), AMG 9810 (an inhibitor of TRPV1) and AMTB (an inhibitor of TRPM8), to determine if TRPV1 or TRPM8 influence CEC TPMD-Ca^++^ Wvs. BCTC reduced mouse CEC TPMD-Ca^++^ Wv cell number, with no effect on human CECs. AMG 9810 reduced the cell number and under curve area in mouse CECs, but not human CECs, while AMTB actually increased human CEC under area curve with no effect on cell number or mouse CECs. These results indicate a species difference, with mouse, but not human CEC TPMD-Ca^++^ Wvs being influenced by TRPV1. It implies that there is a difference in Ca^++^ channel expression or signaling pathways between mouse and human CECs. These results also demonstrate there is likely no influence of TRPM8 on CEC TPMD-Ca^++^ Wvs. A limitation of this study was the use of only pharmacological agents with no genetic models to examine the influence of ion channels on TPMD signaling, and additional studies will be required to determine if the other Ca^++^ channels present in CECs have an influence on TPMD-Ca^++^ Wvs.

It is clear from the current study and the literature that TPMDs can result in Ca^++^ Wvs, and that mechanical stressors can initiate TPMDs. Ca^++^ Wvs from different cell types, even if from the same tissue, can rely on different Ca^++^ sources (intracellular and/or extracellular), and can be influenced by different signaling molecules and or channels. In addition, there are clearly species differences in these variables. It is important to recognize different responses between species (in this and other studies) in that they can help to explain differences observed in results of animal versus human studies, and importantly, to recognize potential issues when validating animal models of human disease and developing therapeutics. Regardless of these differences, this study and the previous studies demonstrating TPMD-Ca^++^ Wvs indicate that they are a natural phenomenon that likely lead to amplified responses to survivable plasma membrane damage of just a single cell. The specific physiological consequences of CEC or CSC TPMDs have yet to be elucidated. Given that gentle eye rubbing creates TPMDs in both CEC and corneal keratocytes, it is likely that TPMDs play a role in routine homeostasis of the cornea. This study demonstrates that routine eye rubbing can trigger TPMDs and TPMD-Ca^++^ Wvs, leading to calcium influx into CECs and keratocytes and likely downstream calcium-initiated events such as shifts in actin dynamics and cell motility, proliferation and transcription. These calcium-initiated events could affect routine epithelial cell migration and turnover, and in the case of keratocytes, collagen turnover. In addition, there is a strong correlation between eye rubbing and keratoconus as reviewed in references [30, 31], although the precise mechanism of how eye rubbing can lead or contribute to the etiology of keratoconus is unknown. It is possible that this newly discovered TPMD-related Ca^++^ signaling pathway present in corneal epithelial cells and keratocytes after eye rubbing is involved.

In cases where the epithelium is compromised, such as diabetic keratitis and dry eye, the signal amplification carried by TPMD-Ca^++^ Wvs could lead to apoptosis and other forms of programmed cell death, and could affect corneal nerves. Blockade of TRPV1 signaling by ocular topical administration was previously shown to decrease the severity of dry eye and other ocular surface inflammatory disorders in mice [32, 33]. In addition, TRPV1 has been linked to the corneal epithelial wound healing response [34]. These effects could be related to the influence of TRPV1 on TPMD-Ca^++^ Wvs.

## Conclusions

This study demonstrates TPMDs and TPMD-Ca^++^ Wvs in human and mouse *in vitro* and *ex vivo* CECs, and in *in vivo* mouse CECs. They also demonstrate that CEC TPMDs can occur from a simple mechanical loading event such as eye rubbing. This study also demonstrates that intracellular Ca^++^ is the primary source of Ca^++^ for CEC TPMD-Ca^++^ Wvs. It is likely that the TRPV1 channel is involved in initiating or proliferating mouse, but not human CEC TPMD-Ca^++^ Wvs, while TRPM8, ATP and gap junctions play little to no role. In addition, there are cell type differences in the properties of TPMD-Ca^++^ Wvs, including different cell types from the same tissue, along with species differences.

## Supporting information

S1 Fig Primary mouse corneal epithelial cell calcium wave video, DMEM, 20X objective. Still photos in Fig 1A–1C captured from this video. Circle highlights the TPMD target on the source cell.
(ZIP)

S2 Fig Primary human corneal epithelial cell calcium wave video, DMEM, 20X objective. Still photos in Fig 1D–1F captured from this video. Circle highlights the TPMD target on the source cell.
(ZIP)

S3 Fig *Ex vivo* mouse corneal epithelial cell calcium wave video, DMEM, 20X objective. Still photos in Fig 1G–1I captured from this video. Circle highlights the TPMD target on the source cell.
(ZIP)

S4 Fig *Ex vivo* human corneal epithelial cell calcium wave video, DMEM, 20X objective. Still photos in Fig 1J–1L captured from this video. Circle highlights the TPMD target on the source cell.
(ZIP)

S5 Fig Multiphoton laser-induced TPMD-Ca^++^ Wv in a living mouse cornea video. Still photos in Fig 2A–2C captured from this video. Circle highlights the TPMD target on the source cell.
(ZIP)

S6 Fig Primary mouse corneal epithelial cell calcium wave following thapsigargin exposure video, 20X objective. Still photos in Fig 6E and 6F captured from this video. Circle highlights the TPMD target on the source cell.
(ZIP)

S7 Fig Primary human corneal epithelial cell calcium wave following thapsigargin exposure video, 20X objective. Still photos in Fig 6G and 6H captured from this video. Circle highlights the TPMD target on the source cell.
(ZIP)

S8 Fig Primary mouse corneal epithelial cell calcium wave following AMG9810 exposure video, 20X objective. Still photos in Fig 6I and 6J captured from this video. Circle highlights the TPMD target on the source cell.
(ZIP)

S9 Fig Primary human corneal epithelial cell calcium wave following AMG9810 exposure video, 20X objective. Still photos in Fig 6K and 6L captured from this video. Circle highlights the TPMD target on the source cell.
(ZIP)
