# Supplementary material for: Transient plasma membrane disruption induced calcium waves in mouse and human corneal epithelial cells
Source: PLoS One. 2024 Apr 17;19(4):e0301495. doi: 10.1371/journal.pone.0301495 (PMC11023258; doi:10.1371/journal.pone.0301495)
Supplement: S2 Fig — Still photos in Fig 1D–1F captured from this video. Circle highlights the TPMD target on the source cell. (ZIP) [file pone.0301495.s002.zip › S2 Fig..pptx]

## Slide 1
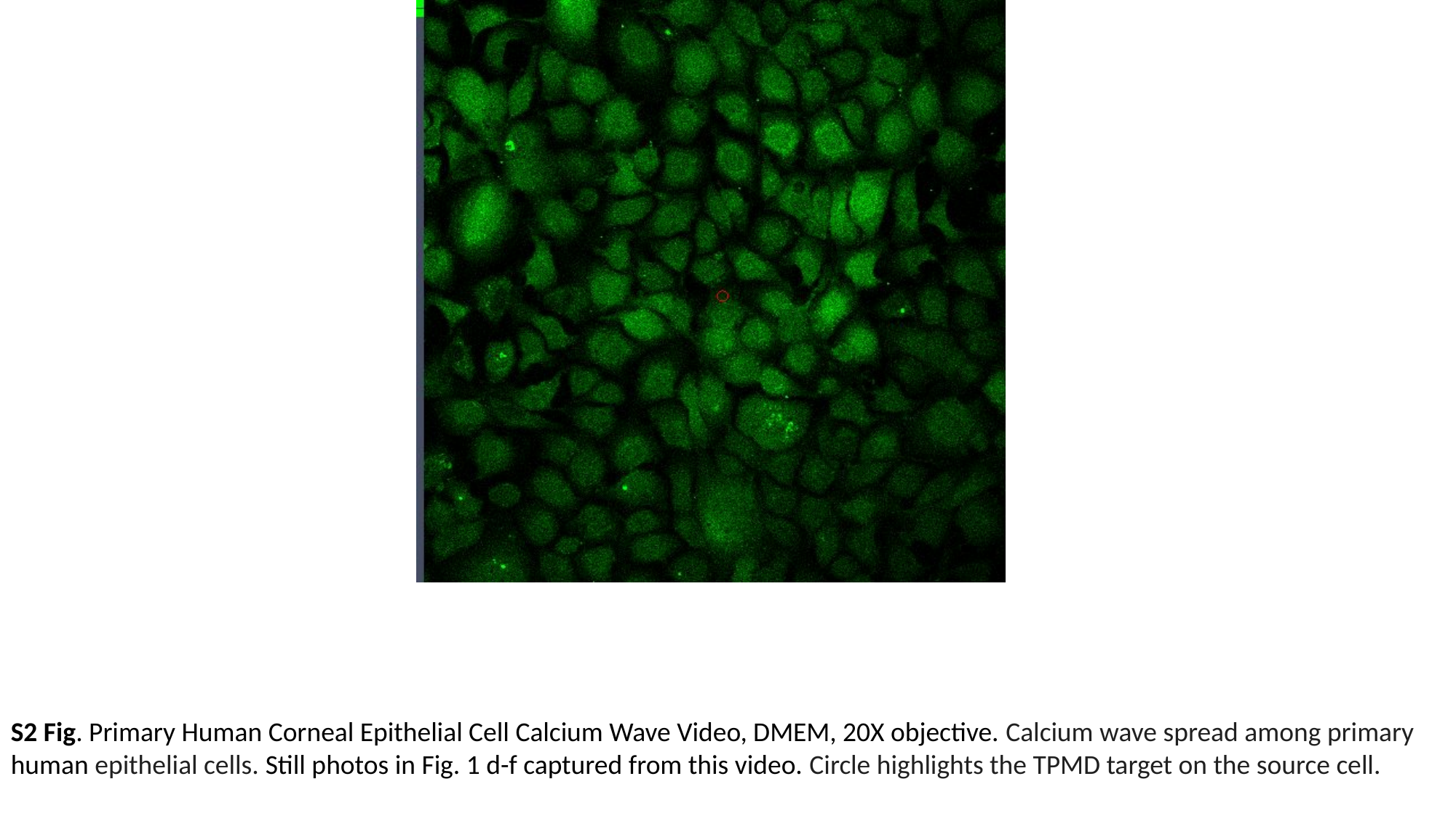

S2 Fig. Primary Human Corneal Epithelial Cell Calcium Wave Video, DMEM, 20X objective. Calcium wave spread among primary human epithelial cells. Still photos in Fig. 1 d-f captured from this video. Circle highlights the TPMD target on the source cell.
