# Supplementary material for: Transient plasma membrane disruption induced calcium waves in mouse and human corneal epithelial cells
Source: PLoS One. 2024 Apr 17;19(4):e0301495. doi: 10.1371/journal.pone.0301495 (PMC11023258; doi:10.1371/journal.pone.0301495)
Supplement: S5 Fig — Still photos in Fig 2A–2C captured from this video. Circle highlights the TPMD target on the source cell. (ZIP) [file pone.0301495.s005.zip › S5 Fig..pptx]

## Slide 1
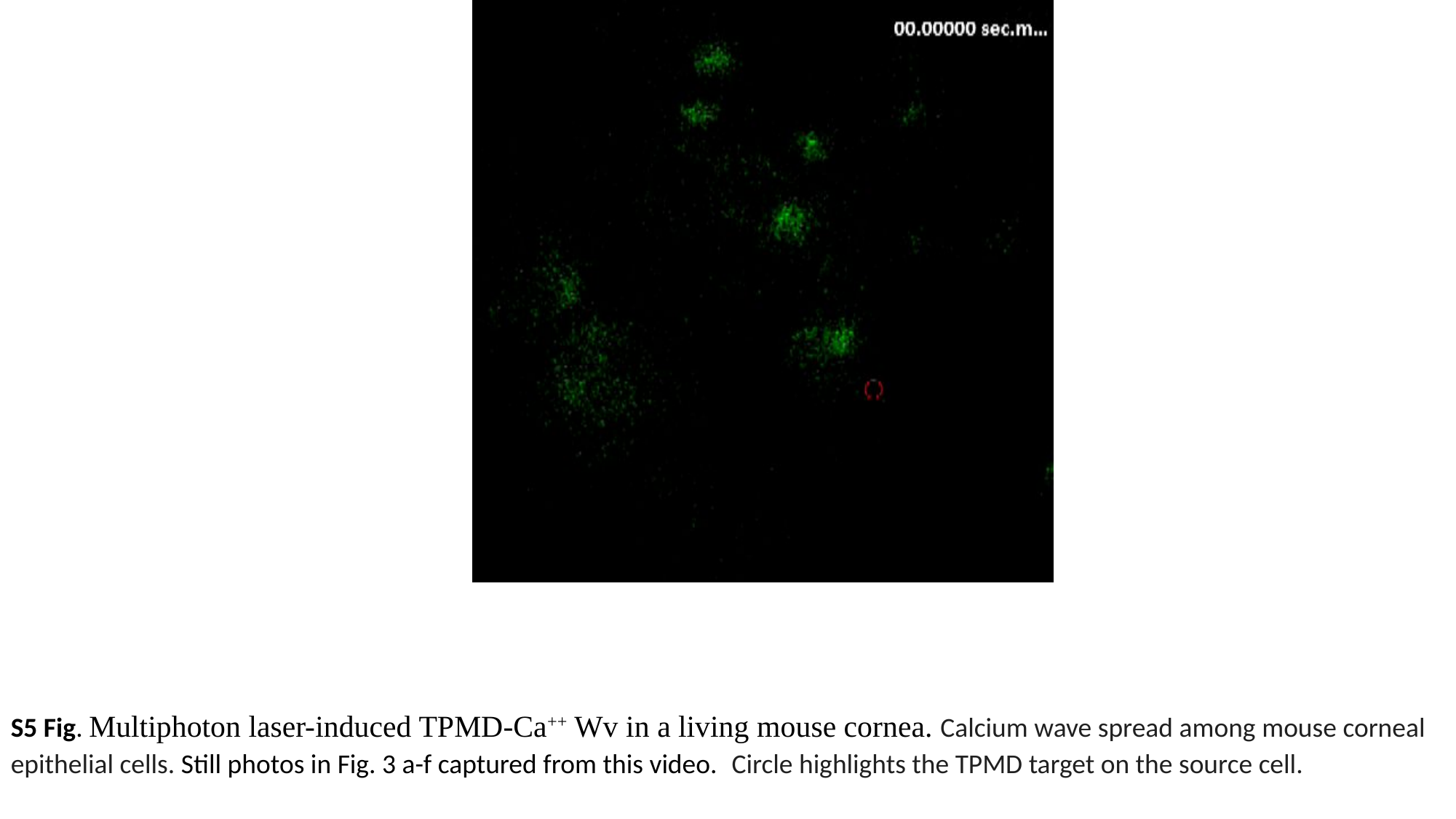

S5 Fig. Multiphoton laser-induced TPMD-Ca++ Wv in a living mouse cornea. Calcium wave spread among mouse corneal epithelial cells. Still photos in Fig. 3 a-f captured from this video. Circle highlights the TPMD target on the source cell.
