# Supplementary material for: Transient plasma membrane disruption induced calcium waves in mouse and human corneal epithelial cells
Source: PLoS One. 2024 Apr 17;19(4):e0301495. doi: 10.1371/journal.pone.0301495 (PMC11023258; doi:10.1371/journal.pone.0301495)
Supplement: S9 Fig — Still photos in Fig 6K and 6L captured from this video. Circle highlights the TPMD target on the source cell. (ZIP) [file pone.0301495.s009.zip › S9 Fig..pptx]

## Slide 1
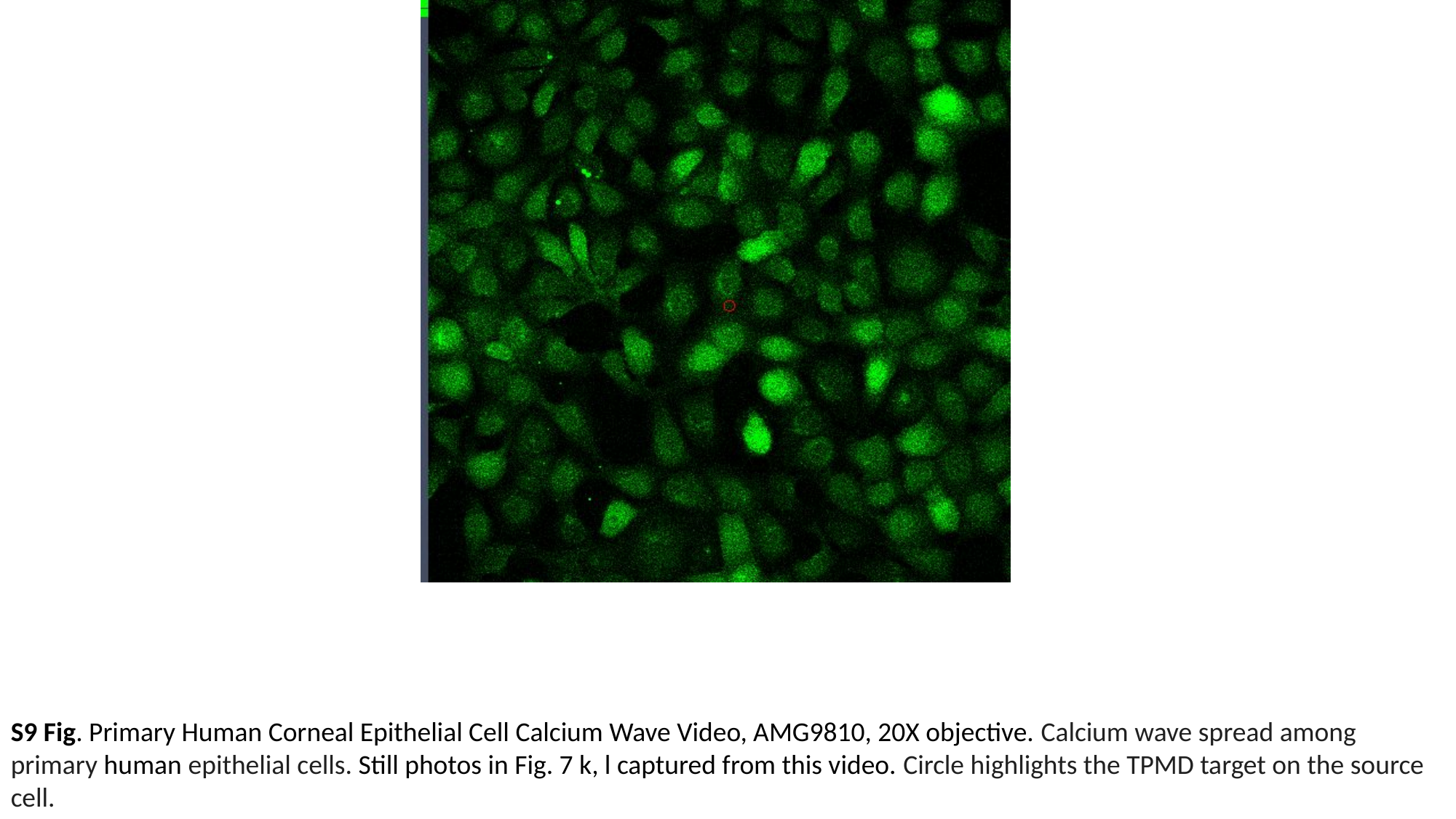

S9 Fig. Primary Human Corneal Epithelial Cell Calcium Wave Video, AMG9810, 20X objective. Calcium wave spread among primary human epithelial cells. Still photos in Fig. 7 k, l captured from this video. Circle highlights the TPMD target on the source cell.
